# Plectin-1 Targeted AAV Vector for the Molecular Imaging of Pancreatic Cancer

**DOI:** 10.3389/fonc.2013.00084

**Published:** 2013-04-18

**Authors:** Prasad R. Konkalmatt, Defeng Deng, Stephanie Thomas, Michael T. Wu, Craig D. Logsdon, Brent A. French, Kimberly A. Kelly

**Affiliations:** ^1^Department of Biomedical Engineering, University of VirginiaCharlottesville, VA, USA; ^2^Department of Cancer Biology, University of Texas MD Anderson Cancer CenterHouston, TX, USA; ^3^Department of Medicine, University of VirginiaCharlottesville, VA, USA; ^4^Department of Radiology, University of VirginiaCharlottesville, VA, USA; ^5^Robert M. Berne Cardiovascular Research Center, University of VirginiaCharlottesville, VA, USA

**Keywords:** AAV, pancreatic cancer, gene therapy, targeted gene delivery, capsid modification, phage display

## Abstract

Pancreatic ductal adenocarcinoma (PDAC) is highly malignant disease that is the fourth leading cause of cancer-related death in the US. Gene therapy using AAV vectors to selectively deliver genes to PDAC cells is an attractive treatment option for pancreatic cancer. However, most AAV serotypes display a broad spectrum of tissue tropism and none of the existing serotypes specifically target PDAC cells. This study tests the hypothesis that AAV2 can be genetically re-engineered to specifically target PDAC cells by modifying the capsid surface to display a peptide that has previously been shown to bind plectin-1. Toward this end, a Plectin-1 Targeting Peptide (PTP) was inserted into the loop IV region of the AAV2 capsid, and the resulting capsid (AAV-PTP) was used in a series of *in vitro* and *in vivo* experiments. *In vitro*, AAV-PTP was found to target all five human PDAC cell lines tested (PANC-1, MIA PaCa-2, HPAC, MPanc-96, and BxPC-3) preferentially over two non-neoplastic human pancreatic cell lines (human pancreatic ductal epithelial and human pancreatic stellate cells). *In vivo*, mice bearing subcutaneous tumor xenografts were generated using the PANC-1 cell line. Once tumors reached a size of ∼1–2 mm in diameter, the mice were injected intravenously with luciferase reporter vectors packaged in the either AAV-PTP or wild type AAV2 capsids. Luciferase expression was then monitored by bioluminescence imaging on days 3, 7, and 14 after vector injection. The results indicate that the AAV-PTP capsid displays a 37-fold preference for PANC-1 tumor xenographs over liver and other tissues; whereas the wild type AAV2 capsid displays a complementary preference for liver over tumors and other tissues. Together, these results establish proof-of-principle for the ability of PTP-modified AAV capsids to selectively target gene delivery to PDAC cells *in vivo*, which opens promising new avenues for the early detection, diagnosis, and treatment of pancreatic cancer.

## Introduction

Pancreatic ductal adenocarcinoma (PDAC) is the fourth leading cause of cancer-related death in the United States. PDAC shows a rapid clinical course, with a median survival of 6 months and a 5-year survival rate of only 3% (Geer and Brennan, 1993; Hezel et al., 2006). The high mortality rate in PDAC patients is linked to the nature of underlying tumor biology. PDAC is highly malignant, metastasizes extensively, and has late onset of clinical symptoms. As pancreatic cancer is highly resistant to chemotherapy and radiotherapy, the only potential cure currently available is complete surgical resection of the tumor. However, surgery is possible only in 10–20% of the patients due to extensive invasion of surrounding structures at the time of diagnosis. Furthermore, complete resection of the primary as well as secondary tumors is rarely achieved (Hezel et al., 2006). Therefore, novel and more effective treatments are needed to improve the survival of pancreatic cancer patients. Targeted gene therapy using an AAV vector designed to selectively deliver therapeutic genes is an attractive and efficient option to treat pancreatic cancer.

With rapid advances in the understanding of vector biology, improved production of high titer stocks, and the discovery of highly efficient new serotypes, AAV vectors have become one of the most promising gene delivery systems currently available. Recombinant AAV vectors transduce a wide variety of tissues *in vivo*. Engineered AAV vectors typically do not integrate into the host genome, but rather exist as relatively stable episomes that are able to provide long-term gene expression. Importantly, AAV vectors provoke minimal immune responses in the host and are almost entirely non-cytotoxic (Hernandez et al., 1999). However, the ability to transduce a wide range of tissues can be a disadvantage when the objective is to target specific cell types for *in vivo* gene therapy. These considerations emphasize the need for the development of AAV vectors that can be used to target specific cells and tissues, even after systemic (intravenous) administration.

In order to render AAV vectors tissue-specific, one can use genetic engineering techniques to express a ligand on the surface of the viral capsid that recognizes a receptor specifically expressed in the target tissue. This alteration is able to change the promiscuous tropism of the wild type AAV to a vector with a singular specificity (Girod et al., 1999; Grifman et al., 2001; Nicklin et al., 2001, 2003; Reynolds et al., 2001; Work et al., 2004). Like other viruses, AAV infects cells by attaching to specific cell surface receptors. In the case of AAV2, heparan sulfate proteoglycan (HSPG) serves as a primary receptor (Summerford and Samulski, 1998; Walters et al., 2004) while FGFR1 (Qing et al., 1999) and αvβ5 integrin (Summerford et al., 1999) serve as co-receptors. The broad tropism of AAV2 is due to the fact that HSPG is present on a wide variety of cell types. Further, mutational analyses of the AAV capsid (cap) proteins have mapped the HSPG binding sites to two regions: between amino acids 509–522 and 561–591 (Wu et al., 2000; Kern et al., 2003). Sequence alignment of AAV with canine parvovirus for which the crystal structure is known, predicted that these two regions form loop III and loop IV of a beta barrel structure (Girod et al., 1999; Grifman et al., 2001). By replacing the loop IV region of AAV structural protein with specific targeting peptides, AAV vectors were constructed that target a number of cell types that were otherwise not permissive for AAV2, including: mouse melanoma cells (Girod et al., 1999), Kaposi sarcoma and rhabdomyosarcoma (Grifman et al., 2001), and HUVECs (Nicklin et al., 2003; Work et al., 2006). These results demonstrate that it is possible to develop AAV vectors targeted to specific receptors of choice for use in gene therapy experiments *in vivo*.

A critical step in developing receptor-targeted vectors is identifying a unique peptide ligand that specifically binds to the target tissue when inserted into the context of the AAV capsid. The choice of ligand to insert into the AAV capsid for the development of a receptor-targeted vector relies on identifying biological peptides with affinity for the specific cell/tissue type of interest. Several groups have applied phage display technology to select targeting peptides out of combinatorial libraries, and have spliced these peptides into the AAV capsid to successfully transduce target cells that were otherwise not permissive for AAV (Girod et al., 1999; Grifman et al., 2001; Nicklin et al., 2001). Recently, Kelly et al. (2008), used phage display on early passage PDAC cell lines isolated from relevant mouse models to identify peptides that distinguish both human and murine PDAC cells from normal pancreatic ductal cells *in vitro* and *in vivo*. Their study showed that a bacteriophage clone displaying peptide 27 (Plectin-1 Targeting Peptide, PTP) was highly specific for human PDAC cells. Further, its target: Plectin-1 (*Plec1*) was subsequently found to be expressed on all PDAC specimens examined (Bausch et al., 2011).

Based on these observations, we hypothesized that an AAV2 capsid bearing PTP inserted into the loop IV region would target it to PDAC cells *in vivo* following systemic administration. In the present study, as proof-of-principle we demonstrate that the genetically modified AAV2 vector bearing PTP peptide preferentially recognized PDAC cells *in vitro* and *in vivo* provided targeted gene delivery in nude mice bearing pancreatic tumor xenografts.

## Materials and Methods

### Plasmids and AAV vectors

The ITR-flanked recombinant AAV2 genome expressing firefly luciferase under the control of the CMV promoter (pAAV-CIL) was derived from pCMV-MCS (Agilent Technologies, Santa Clara, CA, USA) by directionally subcloning the firefly luciferase cDNA (Promega Corp., Madison, WI, USA) between the *Cla*I and *Xba*I sites located downstream of the CMV promoter. The rep/cap plasmid pAAV-PTP was constructed using plasmid pXX2-ΔLoopIV (a gift from Dr. Mathew D. Weitzman, Salk Institute, San Diego, CA, USA). Plasmid pXX2-ΔLoopIV contains AAV rep and cap ORFs with unique *Mlu*I and *Spe*I sites at the loop IV region of the AAV capsid protein designed to allow for the in-frame insertion of peptide-encoding oligonucleotides (Grifman et al., 2001). A synthetic double-stranded oligonucleotide (5′-cgcgtaaaaccctgctgccgaccccga-3′) encoding the heptapeptide KTLLPTP with *Mlu*I and *Spe*I restriction sites at the 5′- and 3′-ends, respectively, was inserted between the *Mlu*I and *Spe*I sites of pXX2-ΔLoopIV as indicated in Table 1. The construction of the plasmid used to generate the reporter vector ACMVLuc has been described previously (Prasad et al., 2011). The AAV plasmid pAAV-sc-CBeGFP harboring a self-complementary AAV genome expressing enhanced green fluorescent protein (eGFP) from the chicken beta-actin promoter was obtained from the Penn Vector Core (Philadelphia, PA, USA). AAV2 viruses were packaged by double transfection and AAV-PTP viruses were packaged by the triple transfection method in 293 cells. Titers for the AAV vectors (viral genomes/ml; vg/ml) were determined by quantitative real-time PCR as described previously (Prasad et al., 2011).

**Table 1:** **Amino acid sequence alignment of wild type AAV2, AAV2ΔLoopIV, and AAV-PTP capsids in the region spanning loop IV**.

Wild type AAV2	TEQYGSVSTNLQRGNR–QAATADVNTQGVLPGMVWQ
AAV2ΔLoopIV	TEQYGSVSTNLQTR–––––DITSDVNTQGVLPGMVWQ
AAV-PTP	TEQYGSVSTNLQTR**KTLLPTP**TSDVNTQGVLPGMVWQ

*The insertion of the Plectin-1 Targeting Peptide (PTP) is indicated in bold characters*.

### Cell lines

Human pancreatic cancer (PDAC) cell lines (PANC-1, MIA PaCa-2, HPAC, MPanc-96, and BxPC-3) were purchased from ATCC. The immortalized human pancreatic ductal epithelial (HPDE) cell line was a kind gift from Dr. Ming-Sound Tsao (University of Toronto, Toronto, ON, Canada). Immortalized human pancreatic stellate cells (hPSC) were developed at the MD Anderson Cancer Center and have been described previously (Hwang et al., 2008). PDAC cells, 293-T and hPSC cell lines were maintained in 10% FBS/DMEM at 37°C in a humidified atmosphere of 5% CO_2_. HPDE cells were maintained in a specialized growth medium composed of keratinocyte serum free medium supplemented with EGF and bovine pituitary extract (Liu et al., 1998).

### *In vitro* bioluminescence imaging

All cells were trypsinized (0.05% TE) and plated into 24-well plates at a density of ∼50,000 cell/well. Upon reaching ∼50–60% confluence, the cells were transfected with virus-containing medium at a dose of 2.5 × 10^4^ vg/cell, in sets of four to eight wells with either blank control (vehicle), or the CMV-driven luciferase reporter vector (pAAV-CIL) packaged in the AAV2, AAV2ΔLoopIV, or AAV-PTP capsids. Before imaging at 24–48 h after transfection, the transfection medium was replaced with new medium containing d-luciferin (Caliper Life Sciences, Hopkinton, MA, USA) at a concentration of 150 μg/ml, and imaged using the IVIS Spectrum system (Caliper Life Sciences) with an exposure time of 1 min. Image data were quantified using Living Image software (Caliper Life Sciences) and are presented as the ratio of AAV-PTP to AAV2ΔLoopIV after background subtraction.

### Mouse model and vector administration

Male athymic nude mice at age 7–9 weeks were purchased from NCI (Frederick, MD, USA) and maintained on a 12/12 h light/dark cycle at 24°C and 60% humidity. Mice were injected subcutaneously twice with 1 × 10^6^ PANC-1 cells bilaterally on the back adjacent to shoulder blades. Tumors were allowed to grow for 4 weeks until tumor size reached 1–2 mm in diameter, at which time AAV vectors containing identical reporter genomes (ACMVLuc) packaged in two different capsids (AAV2 or AAV-PTP) were administered. Three of the tumor-bearing mice received the AAV2 vector and four received the AAV-PTP vector. For IV injection, mice were anesthetized with 1–1.2% isoflurane in oxygen. Viral solutions containing 1 × 10^12^ vg/mouse in 50–100 μl of PBS were slowly injected via the jugular vein. All work was performed according to animal protocols approved by the Institutional Animal Care and Use Committee and conformed to the Guide for the Care and Use of Laboratory Animals.

### *In vivo* bioluminescence imaging

Luciferase expression in live mice was non-invasively assessed by *in vivo* bioluminescence imaging using previously reported methods (Wu et al., 2002; Prasad et al., 2007). Animals were anesthetized and maintained on 1–1.2% isoflurane in oxygen. d-luciferin (150 μg/g body weight, Caliper Life Sciences) was administered to mice by intraperitoneal injection. Eight minutes following d-luciferin administration, all mice were imaged using an IVIS 100 imaging system (Caliper Life Sciences). Photons emitted from the mice were collected and integrated for a period of 1 min. Images were processed using Living Image software (Caliper Life Sciences).

### Luciferase activity assay

Luciferase activity in various organs was measured using reagents from Promega Corp. (Madison, WI, USA). Heart, liver, lung, kidney, spleen, gastrocnemius muscle, and tumors were collected from mice after bioluminescence imaging 14 days post vector injection. Protein extracts from the whole organs were prepared as recommended by the manufacturer and protein concentrations were determined using the Bio-Rad D_C_ BCA protein assay kit. Luciferase activity in the protein samples was determined using a FLUOstar Optima micro-plate reader (BMG Labtech, Durham, NC, USA) and expressed as relative light units per mg of protein (RLU/mg protein).

### Immunohistochemistry

Enhanced green fluorescent protein expression in mouse tumors was documented by immunohistochemistry as previously described (Prasad et al., 2011). Two weeks following vector administration (1 × 10^12^ vg/mouse), tumors were collected and fixed in 3.7% paraformaldehyde for 1 h at 4°C. After washing in PBS (three times, 5 min each), tumors were equilibrated with 30% sucrose in PBS overnight and 6 μm cryosections were prepared. The sections were immunostained for eGFP using rabbit polyclonal anti-GFP antibody (Abcam Inc, Cambridge, MA, USA).

### Statistical analysis

Student’s *t*-test was used to evaluate differences between experimental and control groups with *p* < 0.05 considered significant. All data are expressed as mean ± standard error of the mean.

## Results

### AAV-PTP preferentially targets pancreatic cancer cell lines

**Figure 1:**
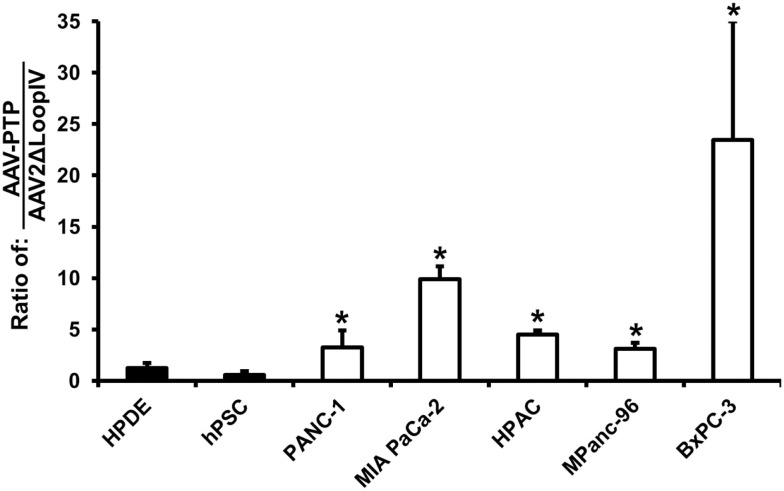
**AAV-PTP preferentially targets human PDAC cells over non-PDAC cells *in vitro***. Two non-neoplastic human cell lines: HPDE (immortalized human pancreatic ductal epithelial cells) and hPSC (immortalized human pancreatic stellate cells) and five human pancreatic cancer cell lines (PANC-1, MIA PaCa-2, HPAC, MPanc-96, and BxPC-3) were either mock-transfected or transfected with identical reporter genomes packaged in the AAV2 capsid, negative control capsid (AAV2ΔLoopIV), or the plectin-1 targeted capsid (AAV-PTP). Luciferase signals were imaged using an IVIS Spectrum and quantified using Living Image software. The results in the graph are presented as the ratio of AAV-PTP to AAV2ΔLoopIV after background subtraction. Asterisks (*) denote *p* < 0.05 vs. the mean of the two non-neoplastic control cell lines (HPDE and hPSC).

An *in vitro* analysis of AAV2 capsids displaying the PTP peptide (AAV-PTP) was performed on five human pancreatic cancer lines (PANC-1, MIA PaCa-2, HPAC, MPanc-96, and BxPC-3). For comparison, two non-neoplastic control cell lines were also included consisting of immortalized HPDE cells and immortalized hPSC. The AAV viruses all contained the same CMV-driven luciferase reporter genome packaged into three different capsids (AAV2, AAV2ΔLoopIV, or AAV-PTP). The primary virus stocks were titered and applied to the PDAC cell lines and non-neoplastic control cell lines. Compared to the control capsid (AAV2ΔLoopIV), the pancreatic cancer-targeted capsid (AAV-PTP) was 4, 13, 6, 4, and 30 times more efficient at the transduction of PDAC cell lines PANC-1, MiaPaCa2, HPAC, MPanc-96, and BxPC-3, respectively; than it was at transducing the non-neoplastic cell lines (HPDE and hPSC, Figure 1). However, this increase in selectivity was associated with a modest loss in overall gene transfer efficiency, since the absolute levels of light production from AAV2 in the neoplastic human cell lines was approximately 4.4-fold higher than AAV-PTP in those same neoplastic cells (data not shown). These results show that the AAV2 vector bearing the PTP peptide preferentially transduces and expresses recombinant genes in human PDAC cell lines over non-neoplastic human pancreatic cell lines, albeit with some loss in overall efficiency relative to AAV2.

### AAV-PTP selectively delivers genes to pancreatic tumors

**Figure 2:**
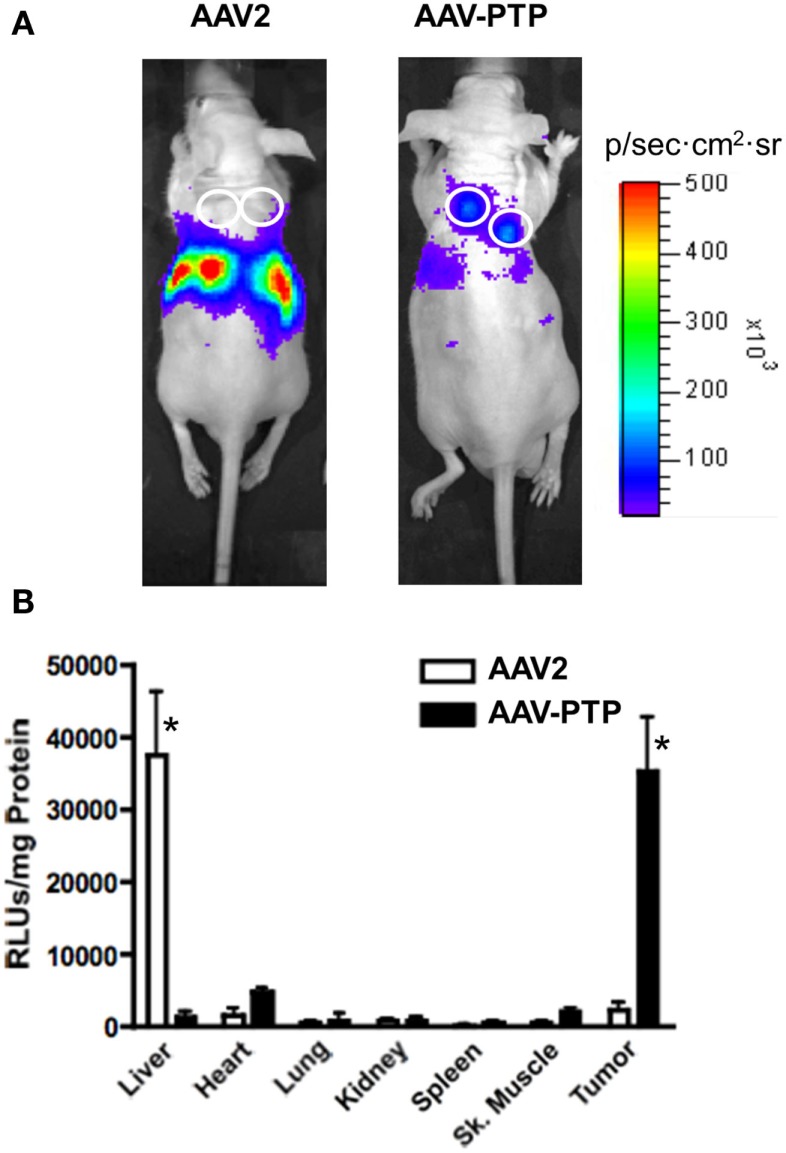
**AAV-PTP selectively targets PDAC tumors *in vivo***. **(A)** Bioluminescence imaging of mice bearing PANC-1 tumor xenografts. Nude mice bearing PANC-1 tumor xenografts (seven total) were injected intravenously with a single dose (1 × 10^12^ vg per mouse) of AAV2 (*n* = 3) or AAV-PTP (*n* = 4) carrying the ACMVLuc reporter vector genome. Following vector administration, luciferase expression was monitored on days 3, 7, and 14 post-injection by non-invasive *in vivo* bioluminescence imaging. Bioluminescence images acquired on day 14 following vector administration are shown. White circles show the sites of tumor cell injections. **(B)** Luciferase activity assay showing tumor-targeted gene delivery. Two weeks following vector administration, the mice described above were euthanized and a panel of tissues (Liver, Heart, Lung, Kidney, Spleen, Skeletal (Sk.) muscle, and Tumor) was collected for quantitative luciferase determinations. Luciferase assays were performed using the *in vitro* luciferase assay kit (Promega Corporation) on protein extracts prepared as per manufacturer’s guidelines. Luciferase activities are reported as relative light units per mg tissue (RLUs/mg tissue). Asterisks (*) denote *p* < 0.05 vs. any other tissue harvested from the same group (AAV2 or AAV-PTP).

In order to test whether the AAV-PTP vector could deliver genes selectively to pancreatic tumors *in vivo*, we used PANC-1 cells to generate mice bearing subcutaneous tumor xenografts. When the tumors reached a size of ∼1–2 mm in diameter (∼4 weeks after inoculation), the mice were injected with AAV-PTP or wild type AAV2 capsids containing identical vector genomes that express luciferase from the CMV promoter. Luciferase expression was monitored by *in vivo* bioluminescence imaging on day 3, 7, and 14 after vector injection. At every time point tested, luciferase expression was predominantly found around the abdominal cavity in mice injected with wild type AAV2 (Figure 2A, left). However, in mice injected with the AAV-PTP capsid, luciferase expression was found predominantly in the tumors (Figure 2A, right) with only minimal expression in non-tumor regions.

To confirm selective gene delivery to the tumors by AAV-PTP, a panel of organs was collected 14 days following vector injection and luciferase activity was measured in the protein extracts. Luciferase assays on protein extracts showed that in mice injected with wild type AAV2 capsids, luciferase activity was predominantly present in the liver as compared to other organs (Figure 2B). On the other hand, in mice injected with AAV-PTP capsids, luciferase activity was 37-fold higher in the tumors as compared to that in liver. Transduction of tumor cells was 24-fold higher with AAV-PTP than with wild type AAV2 capsids. Only low levels of transduction were noted with either wild type AAV2 or AAV-PTP capsids in other organs (heart, lung, kidney, spleen, and skeletal muscle). These results indicate that AAV2 capsids bearing the PTP peptide selectively deliver genes to pancreatic cancer cells in mice bearing pancreatic tumor xenografts.

**Figure 3:**
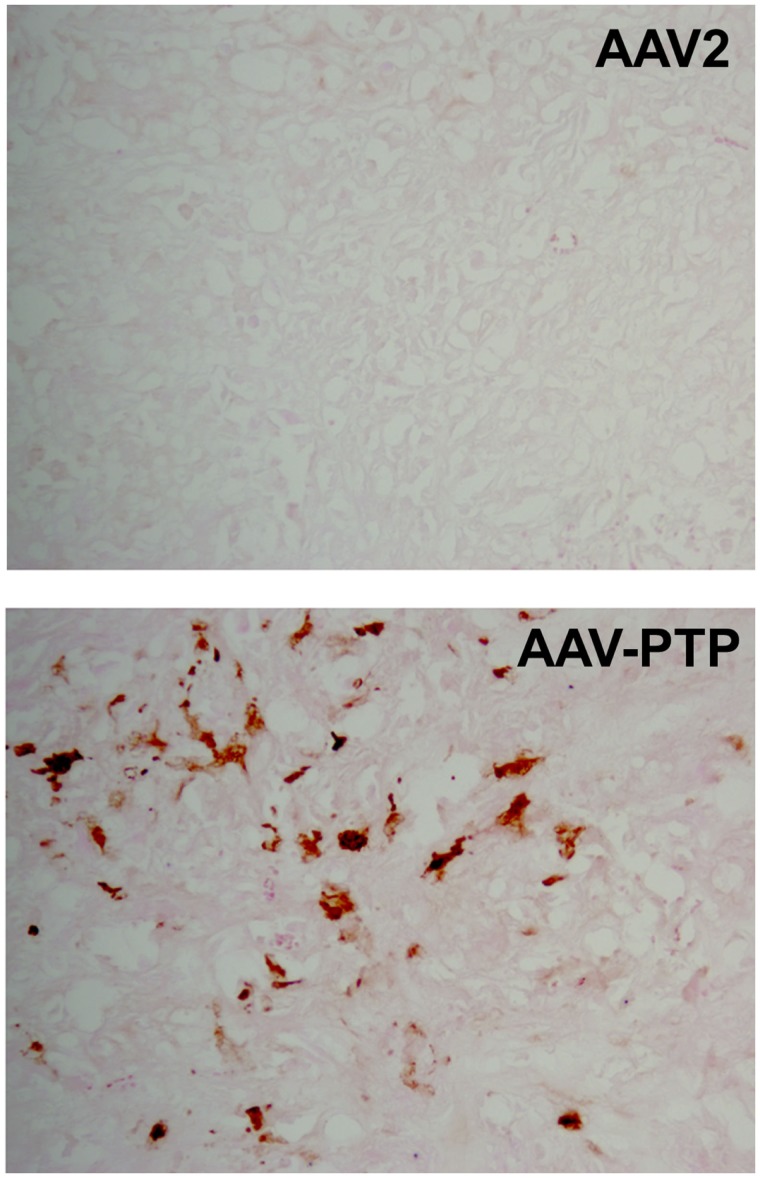
**Distribution of AAV-PTP targeted PANC-1 cells in tumor xenografts**. Immunohistochemistry for eGFP was performed on tumor sections to characterize the distribution of targeted gene delivery. Nude mice bearing PANC-1 tumor xenografts were injected intravenously with a single dose of AAV-PTP capsid carrying the sc-CBeGFP reporter vector genome (1 × 10^12^ vg per mouse). Two weeks following vector administration, cryosections of tumors were prepared and processed for eGFP detection by immunohistochemistry. PANC-1 cells expressing eGFP were stained golden-brown in color by the chromogen substrate (diaminobenzidine).

To test the distribution of gene expression within tumor xenografts, we used an scAAV vector harboring eGFP cDNA under the control of the chicken β-actin promoter (pAAV-sc-CBeGFP). We injected 1 × 10^12^ vg/mouse of either AAV2 or AAV-PTP capsids carrying the AAV-sc-CBeGFP genome into mice bearing PANC-1 xenografts. Two weeks following vector injection, tumors were collected and eGFP expression was detected by immunohistochemical analysis. Results indicated that eGFP expression was evenly distributed throughout the tumor (Figure 3).

## Discussion

Our results demonstrate that replacing the loop IV region of the AAV2 capsid with a peptide that recognizes plectin-1 facilitates selective gene delivery to PDAC cells. This is the first demonstration of a viral vector with pancreatic cancer cell specificity. The Plectin-1 Targeted Peptide (PTP) modified AAV2 capsid was shown to localize to tumor xenografts in mice indicating that the natural tropism of the AAV2 capsid for hepatocytes was dramatically altered. Replacing the loop IV region of AAV2 with PTP did not adversely affect the generation of viral capsids, as evidenced by titers comparable to wild type AAV2. We routinely generated recombinant vectors with titers exceeding 5 × 10^12^ vg/ml, indicating that the insertion of the PTP heptapeptide was well tolerated for efficient packaging of viral particles.

*In vitro* experiments comparing neoplastic and non-neoplastic cell lines showed that AAV-PTP preferentially transduced all 5 PDAC cell lines tested (PANC-1, MIA PaCa-2, HPAC, MPanc-96, and BxPC-3), but did not efficiently transduce two non-neoplastic pancreatic cell types (HPDEs and hPSCs, see Figure 1). Besides providing a foundation for PDAC imaging and neoplasia-targeted suicide gene therapy, this differential in transduction efficiency may prove valuable in basic science studies aimed at dissecting out the relative contributions of neoplastic PDAC cells and non-neoplastic pancreatic cells (e.g., pancreatic stellate cells) in the formation of pancreatic tumors. The PTP peptide was identified by phage display on primary pancreatic cancer cell lines and its binding partner (plectin-1) was found on all primary and metastasized PDAC specimens tested to date (17). Further, the absence of plectin-1 on the plasma membranes of cells in most normal tissues (17) further enhances the ability of PTP to selectively target PDAC cells.

The *in vitro* studies were complemented by *in vivo* studies undertaken to determine whether AAV-PTP can selectively deliver genes to pancreatic tumor cells in mice as proof-of-principle and as an initial step toward gene therapy. We choose plectin-1 positive PANC-1 cells to generate subcutaneous tumor xenografts in mice. Mice bearing PANC-1 tumor xenografts were injected with a luciferase reporter vector packaged in either wild type AAV2 or AAV-PTP capsids. Bioluminescence imaging for luciferase expression showed that, in mice injected with wild type AAV2 capsids, light output was detected in the abdominal cavity, whereas in mice injected with AAV-PTP capsids, light output was detected almost exclusively in the tumors (Figure 2A) with little or no signal originating from the region of the healthy pancreas. These results indicate that the wild type AAV2 capsid preferentially transduced liver hepatocytes whereas the AAV-PTP capsid efficiently transduced tumor cells. Luciferase activity assays performed on protein extracts from various organs collected on day 14 post vector injection demonstrated that luciferase activity in the liver was highest in mice injected with wild type AAV2 capsids. In contrast, in the mice injected with AAV-PTP, luciferase activity was highest in the tumors compared to all other organs. These results show that AAV-PTP capsids selectively transduce pancreatic cancer cells in mouse tumor xenografts with minimal gene expression in other organs including liver following systemic administration. Thus AAV vectors can successfully be modified to deliver genes specifically to pancreatic cancer cells using the cancer cell-specific property of cell surface localization of plectin-1. With further refinements, this approach may hold promise for the early detection, diagnosis and treatment of pancreatic cancer.

## Conflict of Interest Statement

Dr. Kelly is CSO and cofounder of iTi Health, Inc., which has licensed the plectin-1 targeting peptide to develop a SPECT based imaging agent. The other authors have no conflicts of interest to disclose relevant to this manuscript.
